# National Experimental Wellbeing Statistics (NEWS)

**DOI:** 10.23889/ijpds.v9i5.2915

**Published:** 2024-09-18

**Authors:** Jonathan Rothbaum

**Affiliations:** 1 Census Bureau

The NEWS project is improving and expanding estimates of income, poverty, and other measures of economic wellbeing by linking survey, administrative, and commercial data. The initial release addressed biases from unit nonresponse through improved weights, missing income information in survey and administrative data through improved imputation, and misreporting by combining or replacing survey responses with administrative data.

